# Cardiovascular risk after total thyroidectomy in patients with differentiated thyroid carcinoma undergoing levothyroxine replacement monotherapy

**DOI:** 10.3389/fendo.2025.1659736

**Published:** 2025-08-26

**Authors:** Martina Pucci, Marilena Calzolaio, Francesco Ghezzi, Michela Iannotta, Maria Gifuni, Sabrina Ruberto, Francesca Crescenzo, Roberta Esposito, Bernadette Biondi

**Affiliations:** Bernadette Biondi MD Division of Internal Medicine and Cardiovascular Endocrinology. Department of Clinical Medicine and Surgery, University Federico II of Naples, Naples, Italy

**Keywords:** levothyroxine, replacement therapy, differentiated thyroid cancer, cardiovascular risk, thyroidectomized patients, major cardiac events

## Abstract

**Introduction:**

Prospective studies have demonstrated the favorable prognosis of differentiated thyroid cancer, primarily due to its low risk of recurrence and mortality. Considering these favorable outcomes, the most recent ATA guidelines recommend individualizing the degree of TSH suppression to balance the risks and benefits of LT4 therapy based on the aggressiveness of the disease. However, no studies have evaluated the cardiovascular risk in disease-free patients receiving long-term replacement doses of LT4 following the 2016 ATA guidelines.

**Patients and methods:**

This study aimed to evaluate cardiovascular risk in disease-free athyreotic patients with differentiated thyroid cancer according to the 2021 European Society of Cardiology (ESC) guidelines. Only patients without major CV events prior to DTC diagnosis and treated with long-term LT4 therapy after the 2016 ATA guidelines were included. From a larger cohort, 300 disease-free patients who underwent total thyroidectomy—with or without radioiodine (RAI)—were selected and 102 patients were included in this study. The cardiovascular risk was assessed using the ESC 2021 scoring systems: SCORE2, SCORE2-OP, and SCORE2-Diabetes.

**Results:**

Among the 102 patients analyzed in detail, 14 experienced major adverse cardiovascular events (MACE) over a mean follow-up of 12.79 ± 9.13 years post-DTC diagnosis. In patients without MACE, none were classified as having a very high CV risk. A high CV risk was observed in 6% (SCORE2), 38.5% (SCORE2-OP), and 50% (SCORE2-Diabetes) of patients. Moderate CV risk was found in 34% (SCORE2), 38.5% (SCORE2-OP), and 50% (SCORE2-Diabetes), while low risk was recorded in 60% (SCORE2) and 23% (SCORE2-OP).

**Discussion:**

These findings highlight the need for careful cardiovascular monitoring during long-term follow-up in patients with differentiated thyroid cancer. Specific cardiovascular management guidelines are needed in DTC, similar to those available for other cancer populations, to balance the risks and benefits of LT4 therapy and to identify patients at higher cardiovascular risk who may need closer monitoring.

## Introduction

Differentiated thyroid carcinoma (DTC) is expected to become the fourth most frequent malignancy in the United States by 2030 (1). It is characterized by histological and functional features of differentiated thyroid tissue, including the presence of iodine receptors and the synthesis of thyroglobulin (Tg).

Levothyroxine sodium (LT4) monotherapy is the treatment of choice for thyroidectomized patients with DTC, aiming to replace the lack of thyroid hormone secretion and prevent tumor growth from residual or metastatic thyroid tissue (2). Experimental studies have demonstrated that the interaction between TSH- and TSH-receptor on DTC cells upregulates thyroid-specific proteins, such as Tg and sodium-iodide symporter (NIS), and promotes cellular proliferation, supporting the therapeutic potential use of TSH suppression in DTC (2). Consequently, TSH suppression therapy with LT4 after total thyroidectomy and radioiodine ablation was considered mandatory for all DTC patients (2, 3).

However, in recent decades, prospective studies have demonstrated the favorable prognosis of DTC, attributed to its low risk of recurrence and mortality (4–6). Given these favorable outcomes, the recent ATA guidelines recommend individualizing the degree of TSH suppression based on the initial aggressiveness of the thyroid tumor and its behavior during follow-up (5, 6). Updated recommendations for LT4 therapy suggest adjusting the TSH target at the initial evaluation of thyroid cancer based on both the risk of recurrence and the potential adverse effects of TSH suppression (4, 5). Furthermore, during follow-up, a TSH level of <0.1 mIU/L may be appropriate for high-risk patients, 0.1–0.5 mIU/L for those at intermediate risk, and 0.5–2 mIU/L for low-risk patients (4, 5).

The need to balance the risks and benefits of TSH suppression during LT4 therapy arises from evidence, including numerous meta-analyses, indicating that long-term TSH suppression (exogenous subclinical hyperthyroidism) is associated with an adverse cardiovascular prognosis in thyroidectomized patients with DTC (6–8). Alterations in echocardiographic parameters, as well as an increased risk of atrial fibrillation, ischemic stroke, myocardial infarction, and heart failure, have been well documented in some meta-analyses in DTC patients with exogenous subclinical hyperthyroidism during long-term TSH suppression therapy (7). However, conflicting results on the cardiovascular outcomes in DTC patients persist in the literature (8, 9). No studies have assessed the cardiovascular risk in disease-free patients receiving long-term replacement doses of LT4 after the 2016 ATA guidelines (5).

The aim of this study was to assess cardiovascular risk according to the European Society of Cardiology (ESC) 2021 guidelines (10) in a group of thyroidectomized DTC patients without major cardiovascular events prior to their diagnosis.

This retrospective study was performed in a single center at the Department of Internal Medicine, University Federico II of Naples.

## Patients and methods

### Study population

This retrospective, longitudinal, single-center and real-life study

was performed in a single center at the Department of Internal Medicine, University Federico II of Naples.

Disease-free patients were selected from a larger group of patients with DTC who were followed for routine post-operative care. All patients had undergone total thyroidectomy with or without radioiodine (RAI) treatment according to the ATA guidelines (5). All patients were treated with LT4 therapy during the follow-up and were receiving replacement doses after the 2016 guidelines.

The inclusion criteria for selecting patients were:

- Total thyroidectomy for differentiated thyroid cancer.
- Age > 40 years.
- Biochemical euthyroidism during replacement monotherapy with LT4 in the last few years according to the 2016 ATA guidelines.
- Undetectable Tg levels.
- Negative anti-thyroglobulin and anti-peroxidase antibodies.
- Negative neck ultrasonography.
- Absence of comorbid conditions (such as renal, hepatic, neurologic, or psychiatric diseases, autoimmune disorders, gastrointestinal diseases, and malabsorption syndrome).
- No administration of drugs interfering with LT4 absorption or deiodinase activity, and/or affecting the evaluation of TSH and/or thyroid hormones, and/or inducing an altered clearance of LT4.

Exclusion criteria were:

- Pregnant or breastfeeding women.
- Patients with major adverse cardiovascular events (MACE) including stroke, transient ischemic attacks (TIA), acute myocardial infarction, heart failure, and arrhythmias before the diagnosis of DTC.

**Figure 1:**
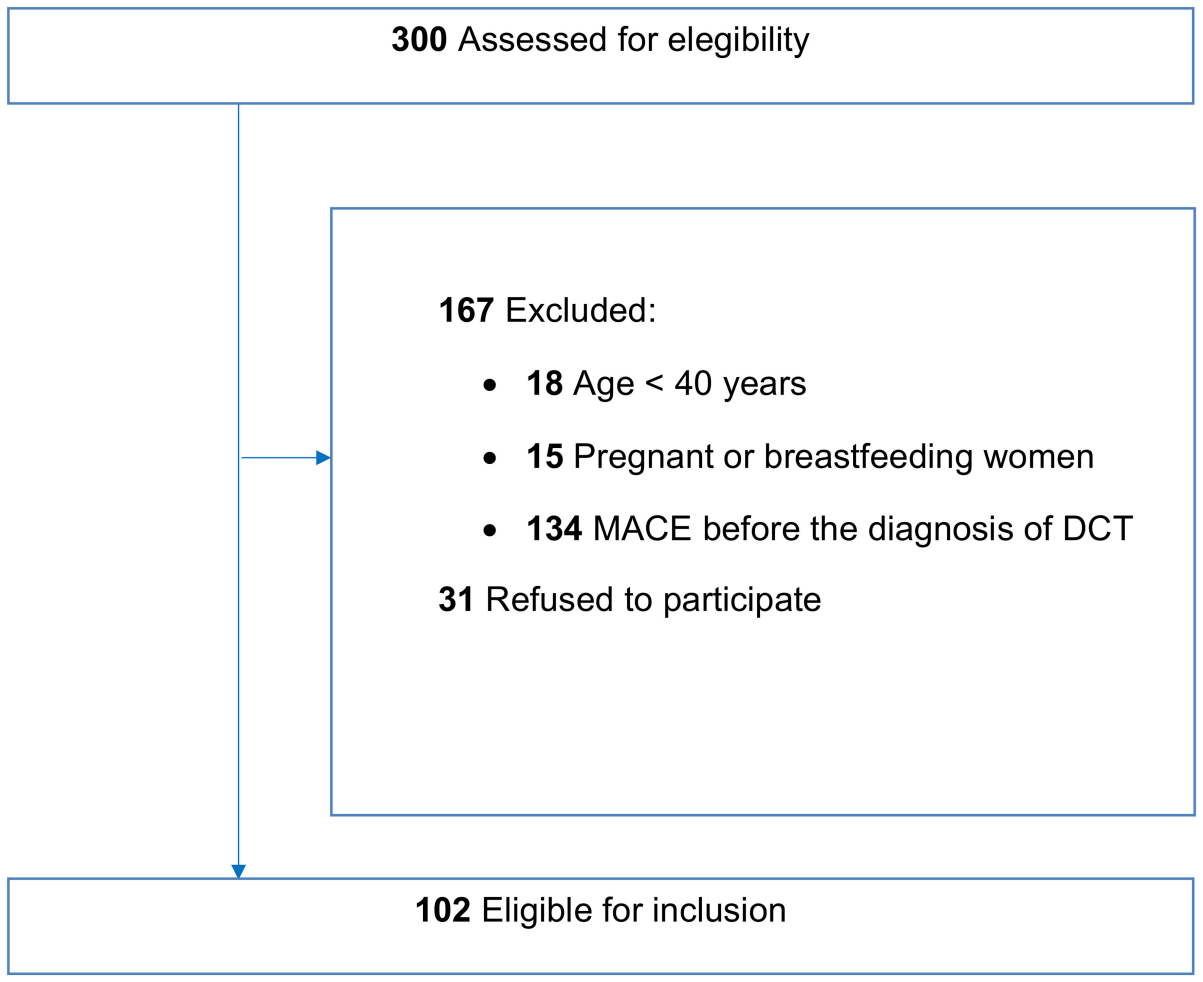
Flowchart describing patient recruitment in the study. A total of 300 patients were assessed for eligibility from a cohort of patients with differentiated thyroid cancer undergoing routine follow-up at our clinic. Of these, 167 patients either did not meet the inclusion criteria and 31 declined to participate, leaving 102 patients eligible for inclusion in the study.

Therefore, among the original 300 patients assessed for eligibility, 167 were excluded and 31 patients refused to participate. As a result, 102 patients were eligible for inclusion in this study after the initial evaluation (**Figure 1**).

### Methods

All the following data were collected from our eligible population of 102 DTC patients:

- Anthropometric characteristics: age, sex, weight and body mass index (BMI).
- Age at diagnosis and duration of follow-up after total thyroidectomy.
- Daily LT4 dose adjusted for body weight.
- Blood test results for TSH, FT3, and FT4 values.
- Cardiovascular risk factors (smoking habits, alcohol consumption status, dyslipidemia, hypertension, body mass index, type II diabetes mellitus).
- Laboratory parameters (total and LDL cholesterol values in mg/dL, blood glucose in mg/dL and glycated hemoglobin (HbA1c) in %, creatinine values in mg/dL, and glomerular filtration rate in mL/min/1.73m²).
- Data on major adverse cardiovascular events (MACE), including strokes, transient ischemic attacks (TIA), myocardial infarction, heart failure and arrhythmia during the follow-up.

The following CV scores from the ESC 2021 guidelines (10) were used to assess cardiovascular risk in our DTC patients:

- **SCORE 2** was applied to patients aged <70 years. To use this score, the following information was required:
  - Sex (male/female)
  - Age (40 to 69 years)
  - Smoking habits (present/absent)
  - Systolic blood pressure (mmHg)
  - Total cholesterol (mg/dL)
  - HDL cholesterol (mg/dL)

- **SCORE 2-OP** was applied to patients aged ≥ 70 years. To use this score, the following information was required:
  - Sex (male/female)
  - Age (70 to 89 years)
  - Smoking habits (present/absent)
  - Systolic blood pressure (mmHg)
  - Total cholesterol (mg/dL)
  - HDL cholesterol (mg/dL)

- **SCORE 2 DIABETES** was considered for patients with type 2 diabetes mellitus. To use this score, the following information was required:
  - Age (40–69 years)
  - Age at diagnosis of type 2 diabetes mellitus
  - Smoking habits (absent/present)
  - Systolic blood pressure (mmHg)
  - Total cholesterol and HDL cholesterol (mg/dL)
  - Glycated hemoglobin (HbA1c) (%)
  - Glomerular filtration rate (mL/min/1.73m²)

The study protocol was approved by the Ethics Committee of the University of Naples Federico II. All patients provided informed consent to the study.

### Statistical analysis

Statistical analysis was performed using IBM SPSS Statistics for Windows v20 (IBM Corp.).

Continuous variables are presented as mean value ± standard deviation. Categorical variables are expressed as absolute numbers and percentages. Differences in continuous variables between the DTC patients who developed MACE and the DTC patients who didn’t develop MACE during the follow up were analyzed using Student’s t-test. Differences in categorical variables between the DTC patients who developed MACE and the DTC patients who didn’t developed MACE during follow up were analyzed using Pearson’s chi-square test. The null hypothesis was rejected at 2-tailed p<0.05.

## Results

### Clinical and cardiometabolic overall results

The mean age of the patients was 57.83 ± 11.54 years, with 80.39% of the patients being female (82 females vs. 20 males). The mean duration of follow-up was 12.79 ± 9.13 years. The mean TSH value during LT4 replacement therapy was 1.08 ± 0.72 μU/ml at the last evaluation. The mean daily dose of LT4 was 1.55 ± 0.47 mcg/kg/day. **Table 1** summarizes the clinical and cardiometabolic characteristics of the overall study population (102 DTC patients) who underwent total thyroidectomy according to the guidelines (5).

**Table 1:** Clinical and cardiometabolic characteristics of the overall study population.

Clinical and cardiometabolic characteristics	Hypothyroid patients with DTC undergoing total thyroidectomy (n=102)
Males n (%)	20 (19.61%)
Females n (%)	82 (80.39%)
Age (years)	57.83 ± 11.54
Weight (Kg)	74.98 ± 14.91
TSH (µU/ML)	1.08 ± 0.89
LT4 (mcg/kg/day)	1.55 ± 0.47
Papillary K, n (%)	89 (87.25%)
Follicular K, n (%)	10 (9.80%)
Medullary K, n (%)	2 (1.96%)
Hurtle’s cell K, n (%)	1 (0.98%)
Radioiodine, n (%)	77 (75.49%)
Smoking n (%)	16 (15.69%)
Dyslipidemia n (%)	50 (49.02%)
Arterial hypertension n (%)	51 (50.00%)
Type II diabetes mellitus n (%)	21 (20.59%)
Time since diabetes diagnosis (years)	1.56 ± 5.32
SBP (mmHg)	127.25 ± 14.26
DBP (mmHg)	81.69 ± 10.22
HR (bpm)	71.95 ± 8.88
TSH (mIU/L)	1.08 ± 0.72
LT4 (mcg/kg/day)	1.55 ± 0.47
Total Cholesterol (mg/dl)	178.82 ± 42.36
LDL (mg/dl)	109.97 ± 37.78
HDL (mg/dl)	53.64 ± 16.80
Triglycerides (mg/dl)	99.23 ± 45.89
HbA1c (%)	5.13 ± 0.68
Creatinine (mg/dl)	0.80 ± 0.13
GFR (ml/min/1.73m^2^)	90.01 ± 16.80

SBP, systolic blood pressure; DBP, diastolic blood pressure; HR, heart rate; LDL, low-density lipoprotein; HDL, high-density lipoprotein; GFR, glomerular filtration rate.

After the first evaluation, our DTC patients were divided into two groups

- Patients who developed major adverse cardiovascular events (MACE) during follow-up (14 patients).
- Patients who did not develop MACE during follow-up (n = 88) had their cardiovascular risk stratified using risk scores in accordance with the 2021 ESC guidelines (10).

### Results in patients with MACE during the follow-up

Data analysis showed that, among the 102 patients analyzed, major adverse cardiovascular events (MACE) occurred in 14 patients, during an average follow-up of 12.79 ± 9.13 years after the diagnosis of DTC. The clinical characteristics and cardiometabolic characteristics of the patients who experienced MACE are summarized in **Table 2**. The main risk factors identified in this patient cohort were dyslipidemia (64,29%) and arterial hypertension (64,29%), followed by type 2 diabetes mellitus (35,71%), with a mean disease duration of 1.92 ± 4.14 years.

**Table 2:** Clinical and cardiometabolic characteristics of 14 patients with dtc who developed mace compared to 88 patients with DTC who didn’t developed mace during follow up.

Clinical and cardiometabolic characteristics	DTC patients with mace (n=14)	DTC patients without mace (n=88)	P-value
Male n (%)	4 (28.57%)	16 (18.18%)	0.280
Female n (%)	10 (71.43%)	72 (81.82%)	0.280
Age (years)	62.14 ± 8.89	57.15 ± 11.80	0.133
Weight (Kg)	75.14 ± 12.22	74.95 ± 15.35	0.965
Papillary K, n (%)	11 (78.57%)	78 (88.64%)	0.253
Follicular K, n (%)	2 (14.29%)	8 (9.09%)	0.413
Medullary K, n (%)	1 (7.14%)	1 (1.14%)	0.257
Hurtle’c cell K, n (%)	0 (0%)	1 (1.14%)	0.863
Radioiodine n (%)	13 (92.86%)	64 (72.73%)	0.091
Smoking n (%)	0 (0%)	16 (18.18%)	0.076
Dyslipidemia n (%)	9 (64.29%)	41 (46.59%)	0.173
Arterial hypertension n (%)	9 (64.29%)	42 (47.73%)	0.194
Type II diabetes mellitus n (%)	5 (35.71%)	16 (18.18%)	0.127
Time since diabetes diagnosis (years)	1.92 ± 4.14	1.51 ± 5.50	0.787
SBP (mmHg)	127.50 ± 14.37	127.20 ± 14.33	0.943
DBP (mmHg)	80.00 ± 8.77	81.95 ± 10.45	0.509
HR (bpm)	68.71 ± 7.40	72.47 ± 9.03	0.143
TSH (mIU/L)	1.43 ± 0.65	1.02 ± 0.72	0.051
LT4 (mcg/kg/day)	1.39 ± 0.55	1.58 ± 0.46	0.177
Total Cholesterol (mg/dl)	175.07 ± 38.93	179.42 ± 43.06	0.723
LDL (mg/dl)	105.43 ± 39.30	110.69 ± 37.72	0.631
HDL (mg/dl)	52.79 ± 10.16	53.77 ± 17.66	0.839
Triglycerides (mg/dl)	118.29 ± 65.10	96.19 ± 41.76	0.095
HbA1c (%)	5.56 ± 0.68	5.06 ± 0.66	**0.012**
Creatinine (mg/dl)	0.82 ± 0.12	0.80 ± 0.13	0.550
GFR (ml/min/1.73m^2^)	87.00 ± 12.19	90.49 ± 17.43	0.473

SBP, systolic blood pressure; DBP, diastolic blood pressure; HR, heart rate; LDL, low-density lipoprotein; HDL, high-density lipoprotein; GFR, glomerular filtration rate. Bold indicates significant results.

**Figure 2:**
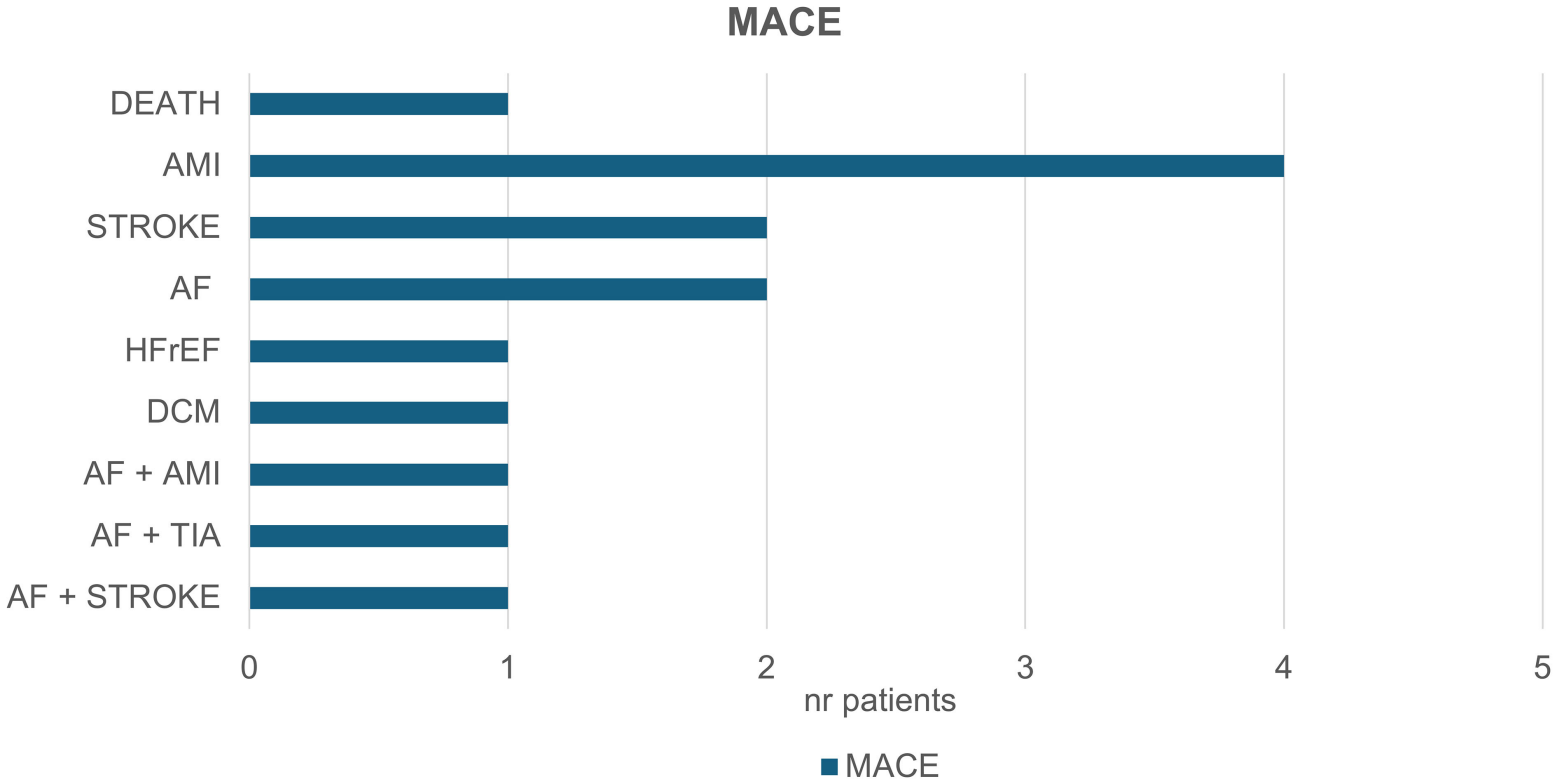
Major adverse cardiovascular events (MACE) in the context of your study. 14 patients experienced the following events: Five patients experienced acute myocardial infarctions (AMI), with one patient subsequently dying. One patient developed dilated cardiomyopathy (DCM) which later required cardiac resynchronization therapy with a defibrillator (CRT-D). One patient developed heart failure with reduced ejection fraction (HFrEF). Atrial fibrillation (AF) was observed in five patients, leading to a transient ischemic attack (TIA) in one case, and to cardioembolic stroke in another. Additionally, two patients suffered a stroke not associated with AF.

The distribution of MACE is shown in **Figure 2**. Acute myocardial infarction (AMI) occurred in five patients, one of whom subsequently died. One patient developed dilated cardiomyopathy (DCM), which progressed to the point of requiring cardiac resynchronization therapy with defibrillator (CRT-D) implantation. Heart failure with reduced ejection fraction (HFrEF) was diagnosed in one patient. Atrial fibrillation (AF) was detected in five patients and was associated with a transient ischemic attack (TIA) in one case and a cardioembolic stroke in another. Additionally, two additional patients experienced strokes not related to AF.

### Results in patients without MACE during the follow-up

**Figure 3:**
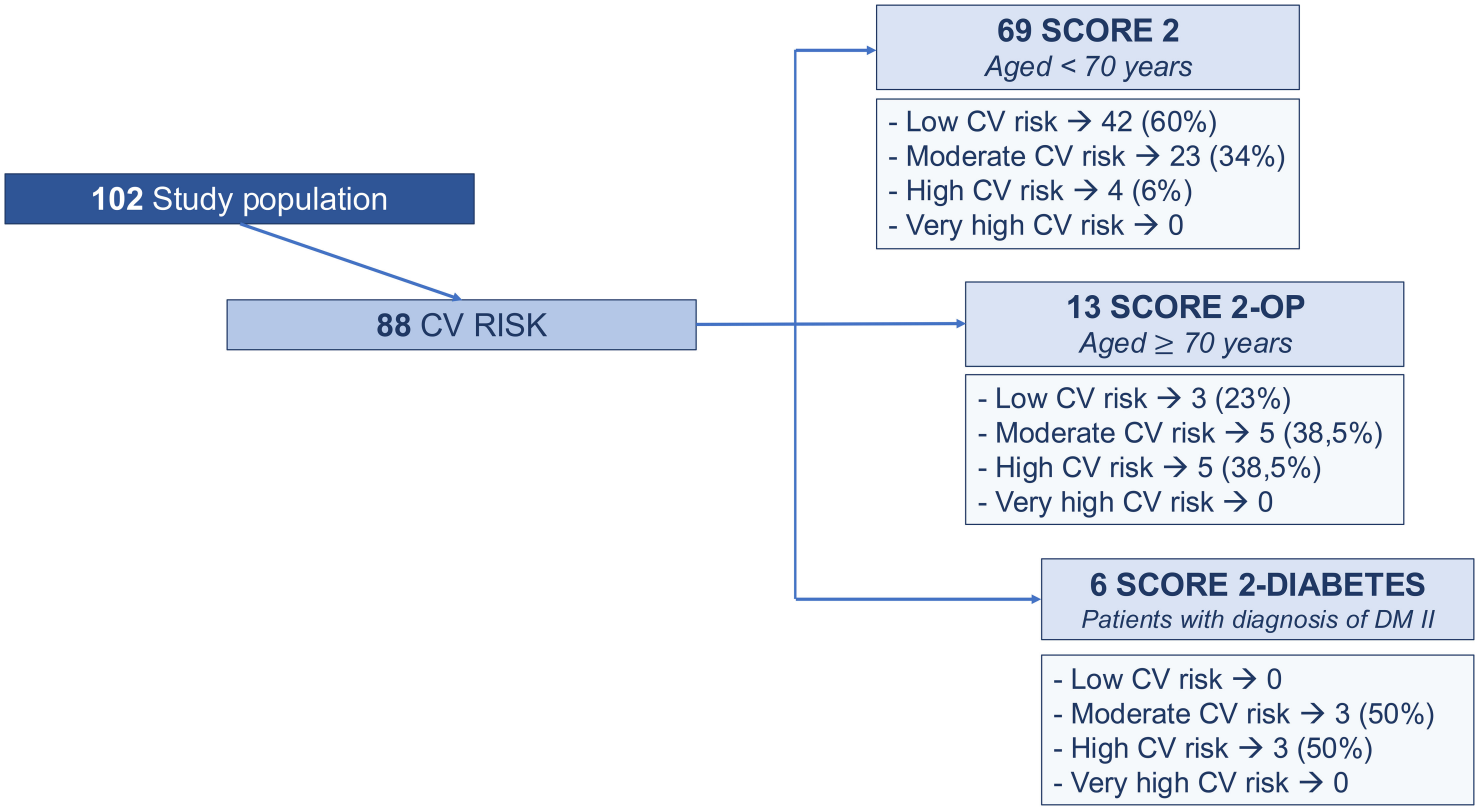
Cardiovascular risk profile assessment based on 2021 ESC guidelines: score 2 (Systematic Coronary Risk Evaluation 2) in patients younger than 70 years, SCORE 2 OP (Systematic Coronary Risk Evaluation 2-Older Persons) in patients older than or equal to 70 years; SCORE 2 Diabetes (Systematic Coronary Risk Evaluation 2-Diabetes) in patients diagnosed with type II diabetes mellitus.

After excluding the 14 patients who developed major adverse cardiovascular events (MACE), the cardiovascular risk profile was assessed in the remaining 88 patients (**Figure 3**). The clinical characteristics and cardiometabolic risk factors of these patients were comparable to those of patients who experienced MACE (**Table 2**). However, HbA1c levels were significantly higher in DTC patients with MACE compared to those without MACE (5.56 ± 0.68 vs 5.06 ± 0.66, p-value 0.012).

Regarding the evaluation of the 2021 ESC -CV score (10), 69 patients aged <70 years were assessed according to the SCORE 2, 13 patients aged ≥ 70 years were evaluated by SCORE 2-OP and 6 patients with type 2 diabetes were assessed by SCORE 2-diabetes (**Figure 3**).

No patients were classified as having a very high cardiovascular (CV) risk. A high CV risk was identified in 6% of patients according to the SCORE2 algorithm, in 38.5% based on the SCORE2-OP model, and in 50% according to the SCORE2-Diabetes algorithm. A moderate CV risk was observed in 34% of patients using SCORE2, 38.5% using SCORE2-OP, and 50% using SCORE2-Diabetes. A low CV risk was found in 60% of patients evaluated with SCORE2 and in 23% of those assessed with SCORE2-OP.

Dyslipidemia, hypertension and smoking habits were the major cardiovascular risk factors in patients with moderate and high risk without MACE.

## Discussion

Patients with differentiated thyroid cancer (DTC) generally have a high disease-specific survival rate (11), however, increased cardiovascular risk factors (7) and adverse cardiovascular outcomes have been reported among thyroid cancer survivors during long-term follow-up. (7–9, 12). Cardiovascular diseases were the leading cause of non-cancer deaths, accounting for 21.3% of all deaths in thyroid cancer patients (12). In the study by Klein Hesselink et al. (13), DTC patients with lower TSH values had an increased risk of cardiovascular mortality. The adjusted relative risk (aRR) was 3.35 (CI 1.66–6.74) for each 10-fold decrease in geometric mean TSH, after adjusting for pre-existing cardiovascular disease or risk factors (13).

The adverse cardiovascular effects of exogenous subclinical hyperthyroidism resulting from long-term TSH suppression were first recognized in 1993, when alterations in cardiac morphology and function, along with an increased risk of atrial arrhythmia, were reported in the context of undetectable serum TSH levels (14). Two meta-analyses of observational studies (7, 15) confirmed these cardiovascular changes, demonstrating higher diastolic blood pressure, heart rate, and left ventricular mass index, as well as reduced early-to-late ventricular filling velocity ratios in DTC patients compared to euthyroid healthy controls during prolonged TSH suppression therapy.

In our study, 14 patients experienced major adverse cardiovascular events (MACE) during follow-up after total thyroidectomy and radioiodine (RAI) therapy. Among our patients with atrial fibrillation (AF), one developed a transient ischemic attack (TIA), and another experienced a cardioembolic stroke. Additionally, two patients suffered a stroke in the absence of AF, supporting the hypothesis that other pathophysiological mechanisms—such as hypercoagulability, systolic hypertension, and increased arterial stiffness—may contribute to the elevated risk of stroke observed in DTC patients.

Some studies and meta-analyses have reported a significantly higher risk of atrial fibrillation (AF) in DTC patients after adjusting for CV risk factors (9, 16). Four studies found that the risk of incident AF was significantly higher in DTC patients compared to cancer-free controls, after adjusting for established risk factors such as age, hypertension, diabetes, and coronary artery disease (7, 16–18). Another study reported a higher prevalence of AF when compared to the general population (19). In two studies, the risk of AF was significantly associated with older age and hypertension (15, 19). Interestingly, the risk of AF was associated with TSH suppression in some reports (17–20). In one study (17), DTC patients had a higher risk of developing AF across all categories of LT4 dosage; the adjusted hazard ratio (aHR) was highest in the highest dosage quartile, which might suggest a dose-response relationship (21), although no correction for body weight was available. Moreover, in another study (19), patients with a TSH <0.1 mIU/L had an increased risk of cardiovascular morbidity (HR: 1.27, CI: 1.03–1.58), mostly attributed to AF (20).

Regarding the risk of stroke, only two studies, including 3,910 DTC patients, examined the risk of stroke in DTC survivors compared to the general population, after adjusting for cardiovascular (CV) risk factors (17, 20). The adjusted relative risk (aRR) for cerebrovascular disease was 1.14 for DTC survivors compared with controls (CI 0.84–1.55) (9).

The Korean National Health Insurance data on DTC patients showed an elevated risk for coronary heart disease (CHD) and ischemic stroke, with hazard ratios (HR) of 1.15 (95% CI: 1.10–1.22) and 1.15 (95% CI: 1.09–1.22), respectively (21). This risk was further increased in patients who received a higher dosage of LT4 (HR: 1.47, 95% CI: 1.34–1.60 for CHD, and HR: 1.56, 95% CI: 1.42–1.72 for ischemic stroke among those who took ≥170 μg/day LT4) (21).

Two studies assessed the risk of ischemic heart disease by comparing DTC patients with controls, after adjusting for cardiovascular (CV) risk factors (17, 20). The pooled adjusted relative risk (aRR) for ischemic heart disease was 0.95 (CI 0.82–1.09), with an I² of 0% (9). Among our patients who experienced major adverse cardiovascular events (MACE) during the follow-up, five suffered acute myocardial infarctions (AMI), with one patient subsequently dying during the follow-up period. These outcomes may be attributed to underlying mechanisms such as systolic hypertension, increased arterial stiffness, dyslipidemia, and diabetes, which are known contributors to cardiovascular risk.

A high and moderate CV risk was observed even in our group of patients without MACE during the follow-up, when assessed by 2021 ESC guidelines (10). Hypertension, diabetes, and dyslipidemia were among the major cardiovascular risk factors observed in our study. Park et al. (22) demonstrated an association between TSH suppression and an increased risk of hypertension and arterial disease among thyroid cancer survivors. In a large population-based cohort in Korea, patients with DTC were more likely to develop type 2 diabetes mellitus (HR, 1.22; 95% CI, 1.08-1.38) and hyperlipidemia (HR, 1.36; 95% CI, 1.24-1.48) compared to matched controls (23). A nonlinear, U-shaped, dose-dependent relationship between LT4 dosage and the risk of type 2 diabetes mellitus was found in DTC patients (P = .021), although the risk of hyperlipidemia was low with high doses of levothyroxine (P = .003). (23). Our results confirm literature data regarding an increased risk of diabetes in patients with DTC. Moreover, interestingly, in our study, patients who developed MACE during the follow-up had higher HbA1c levels compared to those without MACE

It is important to emphasize that our findings were observed in DTC patients receiving long-term L-thyroxine replacement therapy after the 2016 ATA guidelines. These results suggest that cardiovascular risk should be carefully monitored even during the follow-up of patients undergoing L-thyroxine replacement therapy. Regarding the potential mechanisms linked to the increased cardiovascular (CV) risk observed during LT4 replacement therapy in DTC patients, it is possible that the period of overtreatment in the years before the 2016 ATA guidelines could have influenced our results. Overtreatment is frequently observed even among low-risk DTC patients (24) and periods of exogenous hyperthyroidism or hypothyroidism can develop during long-term follow-up of patients receiving replacement doses of L-thyroxine, A recent study suggests that both exogenous subclinical hyperthyroidism and hypothyroidism are associated with an increased risk of cardiovascular mortality (25). Lastly, T3 deficiency has been reported in patients submitted to total thyroidectomy and has been associated with impaired cardiovascular function and dyslipidemia (26, 27).

### Limitations of the current study

One of the most important limits of this study is the lack of age- and sex- match population from the same geographic area without levothyroxine TSH suppressive therapy. The use of the European Society of Cardiology (ESC) guidelines SCORE2, SCORE2-OP, and SCORE2-Diabetes is a methodologically rigorous approach to ensure the validity of our findings. However, these tools are not designed for cancer survivor populations and do not incorporate thyroid-specific or cancer-related parameters. The CV risk in DTC patients is multifactorial, resulting from: classical cardiovascular risk factors (diastolic dysfunction, hypertension, diabetes, dyslipidemia),cancer-related mechanisms (chronic inflammation, oxidative stress, and premature vascular aging) (28), treatment-specific factors (long-term TSH suppression therapy and RAI) and possible alterations in glucose metabolism (29). Some studies have highlighted the limitations of general population–based risk models in DTC survivors and proposed a more nuanced, DTC-adapted tool: the “cardiovascular fingerprint,” which incorporates metabolic, demographic, and treatment-related components (30) Moreover, in other cancer populations, more tailored predictive tools have been developed. For example, the LIFE-CV2 model, recently validated in cancer survivors, provides a dynamic and individualized approach to assessing lifetime cardiovascular risk (31). This underscores the importance of creating dedicated predictive models for thyroid cancer survivors.

## Conclusion

Our findings highlight the importance of considering cardiovascular (CV) risk factors when determining TSH target levels during L-thyroxine replacement therapy in patients with differentiated thyroid cancer (DTC).

There is a clear need for dedicated CV management guidelines in DTC—similar to those available for other cancer populations—to better address and mitigate the increased CV morbidity and mortality observed in these patients. Further studies are needed to evaluate whether the application of future guidelines could help optimize the risk-benefit balance in LT4 dosing and aid in identifying DTC patients with elevated CV risk who may require closer cardiovascular monitoring.

Overweight and smoking habits should be avoided, and physical activity should be recommended for patients with DTC. Additionally, antihypertensive drugs and lipid-lowering medications should be considered for patients with high blood pressure and/or dyslipidemia to reduce the increased CV risk. Beta-blockers can also be considered to reduce elevated heart rates and the risk of atrial arrhythmia (32).

## Data Availability

The raw data supporting the conclusions of this article will be made available by the authors, without undue reservation.
